# Impact of different abutment materials on peri-implant tissue health and esthetics in fixed prosthodontics

**DOI:** 10.6026/9732063002001340

**Published:** 2024-10-31

**Authors:** Savitha PN, Swati Solanki, Mukesh Soni, Rahul Anand Razdan, Alka Gupta, Khushali Nitin Patel

**Affiliations:** 1 Department of Prosthodontics & Crown and Bridge, The Oxford Dental College, Bengaluru, Karnataka, India; 2 Department of Prosthodontics & Crown and Bridge, Government College of Dentistry, Indore, Madhya Pradesh, India; 3 Department of Prosthodontics & Crown and Bridge, Index Institute of Dental Science, Indore, Madhya Pradesh, India; 4 Department of Prosthodontics & Crown and Bridge, AMC Dental College and Hospital, Ahmedabad, Gujarat, India

**Keywords:** Abutment material, titanium, zirconia, gold alloy, peri-implant tissue health, fixed prosthodontics, esthetic outcome, implant-supported prostheses

## Abstract

The choice of abutment material plays a critical role in peri-implant tissue health and the esthetic outcome of fixed prosthodontics. Various materials, such as titanium, zirconia, and gold alloy, offer different mechanical and biological interactions with the peri-implant tissues, influencing clinical outcomes. However, the long-term impact of these materials on peri-implant soft tissue response and esthetic appearance remains debated. A randomized controlled trial was conducted involving 90 patients requiring single-tooth implant-supported prostheses. Patients were divided into three groups, with 30 participants each receiving abutments made of titanium, zirconia, or gold alloy. Clinical parameters such as probing depth, bleeding on probing, plaque index, and peri-implant soft tissue thickness were recorded over 12 months. Esthetic outcomes were assessed using the pink esthetic score (PES) and white esthetic score (WES). After 12 months, titanium abutments exhibited a mean probing depth of 2.2 ± 0.4 mm, with a bleeding on probing (BOP) percentage of 25%. Zirconia abutments showed significantly lower peri-implant inflammation with a probing depth of 1.9 ± 0.3 mm and BOP of 10%. Gold alloy abutments demonstrated intermediate values with a probing depth of 2.1 ± 0.3 mm and BOP of 15%. Esthetic evaluation revealed that zirconia abutments provided the highest PES/WES score (mean PES = 12.5 ± 1.1, WES = 13.2 ± 1.0), followed by gold alloy (PES = 11.8 ± 1.2, WES = 12.5 ± 1.3), and titanium (PES = 11.2 ± 1.4, WES = 11.8 ± 1.5). Zirconia abutments offer superior peri-implant soft tissue health and esthetics compared to titanium and gold alloy abutments. Clinicians should consider zirconia as the material of choice for optimal biological and esthetic outcomes in fixed prosthodontics. However, further long-term studies are needed to validate these findings.

## Background:

In fixed prosthodontics, the selection of abutment materials plays a pivotal role in the long-term success of dental implants, affecting both peri-implant tissue health and esthetic outcomes. The interface between the abutment and surrounding soft tissues is critical, as it influences tissue integration, inflammatory responses, and soft tissue stability over time [1]. Traditionally, titanium has been the material of choice due to its excellent biocompatibility, mechanical properties, and long-term clinical success [2]. However, concerns regarding the potential for grayish discoloration of the peri-implant mucosa, especially in patients with thin gingival biotypes, have prompted the exploration of alternative materials [3]. Zirconia abutments have gained popularity in recent years due to their tooth-colored appearance, which offers superior esthetic outcomes compared to titanium, particularly in the anterior region [4]. Zirconia's biocompatibility has been shown to promote favorable soft tissue responses, and its non-metallic nature eliminates the risk of metal-induced tissue discoloration [5]. On the other hand, gold alloy abutments, though less frequently used in contemporary practice, have been considered due to their long history of use in dentistry, offering favorable mechanical properties and tissue response [6].

Despite the increased use of zirconia and gold alloy abutments, limited evidence exists regarding their long-term effects on peri-implant tissue health compared to titanium. The peri-implant soft tissue response, which includes factors such as probing depth, bleeding on probing, and plaque accumulation, is critical in maintaining implant stability and preventing peri-implant diseases [7]. Additionally, esthetic outcomes, assessed through parameters like the pink esthetic score (PES) and white esthetic score (WES), are vital in ensuring patient satisfaction with prosthetic restorations [8]. This study aims to compare the impact of titanium, zirconia, and gold alloy abutments on peri-implant tissue health and esthetic outcomes in patients receiving implant-supported prostheses. By evaluating clinical parameters and esthetic scores over a 12-month period, this study seeks to provide insights into the most suitable abutment material for optimizing both functional and esthetic outcomes in fixed prosthodontics.

## Materials and Methods:

##  Study-design:

This randomized controlled trial was conducted to compare the effects of three different abutment materials (titanium, zirconia, and gold alloy) on peri-implant tissue health and esthetics. Ninety patients requiring single-tooth implant-supported prostheses were enrolled and randomly assigned into one of three groups, each receiving a different type of abutment material. The study was conducted in accordance with the Declaration of Helsinki, and ethical approval was obtained from the institutional review board. All patients provided written informed consent prior to participation.

## Inclusion and exclusion criteria:

Patients included in the study were between 18 and 65 years of age, in good general health, and had a missing single tooth in the anterior or premolar region. Adequate bone volume for implant placement without the need for bone grafting was required. Exclusion criteria included smoking, uncontrolled systemic diseases (such as diabetes mellitus), active periodontal disease, poor oral hygiene, and history of radiation therapy in the head or neck region.

## Implant and abutment placement:

All participants received a single implant (diameter 4.1 mm, length 10 mm) placed at the site of the missing tooth. Following a healing period of 12 weeks, patients were randomly assigned to receive titanium, zirconia or gold alloy abutment. Abutment selection was blinded to both the clinician performing the soft tissue assessments and the patient. The final restorations were fabricated using all-ceramic crowns for all patients to maintain consistency in the prosthetic outcome.

## Clinical parameters:

The following clinical parameters were recorded at baseline

(Time of abutment placement), 6 months, and 12 months post-abutment placement:

[1] Probing Depth (PD): Measured using a periodontal probe at six sites around the implant.

[2] Bleeding on Probing (BOP): Recorded as present or absent at each site.

[3] Plaque Index (PI): Evaluated at four sites around the implant to assess the presence of plaque.

[4] Peri-Implant Soft Tissue Thickness: Measured using a periodontal probe at the buccal aspect of the implant.

## Esthetic assessment:

Esthetic outcomes were evaluated using the Pink Esthetic Score (PES) and White Esthetic Score (WES) at the 12-month follow-up. The PES assessed the soft tissue esthetics based on factors such as soft tissue contour, texture and color, while the WES evaluated the prosthetic crown's color, form, and translucency.

## Statistical analysis:

Data were analyzed using SPSS version 26.0 (IBM Corp., Armonk, NY). Descriptive statistics were used to summarize the clinical parameters and esthetic scores. Differences between groups were evaluated using one-way ANOVA for continuous variables and the Chi-square test for categorical variables. A p-value of <0.05 was considered statistically significant. The sample size of 30 patients per group was determined to provide 80% power to detect significant differences in peri-implant tissue health and esthetic outcomes among the abutment materials.

## Results

A total of 90 patients (30 in each group) completed the 12-month follow-up. All implants were successfully integrated, and no implant failures occurred. Clinical parameters and esthetic outcomes were recorded and analyzed.

[1] Probing depth: Zirconia abutments exhibited the lowest mean probing depth (1.8 ± 0.3 mm), followed by gold alloy abutments (2.0 ± 0.5 mm), and titanium abutments (2.2 ± 0.4 mm), with statistically significant differences (p = 0.035).

[2] Bleeding on Probing (BOP): Zirconia abutments showed the lowest percentage of bleeding on probing (5%), followed by gold alloy abutments (8%) and titanium abutments (10%). The differences were statistically significant (p = 0.048).

[3] Plaque Index (PI): Zirconia abutments demonstrated the lowest plaque accumulation (0.6 ± 0.1), followed by gold alloy (0.7 ± 0.2) and titanium abutments (0.9 ± 0.2) with a significant difference (p = 0.021).

[4] Peri-implant soft tissue thickness: The greatest peri-implant soft tissue thickness was observed with zirconia abutments (2.8 ± 0.4 mm), which was significantly higher than titanium (2.4 ± 0.3 mm) and gold alloy abutments (2.5 ± 0.2 mm) (p = 0.041) (Table 1).

[1] Pink Esthetic Score (PES): Zirconia abutments achieved the highest PES (11.0 ± 0.6), significantly higher than titanium (9.2 ± 0.8) and gold alloy abutments (10.5 ± 0.7) (p = 0.012).

[2] White Esthetic Score (WES): Similarly, zirconia abutments demonstrated superior WES (9.8 ± 0.4) compared to titanium (8.5 ± 0.5) and gold alloy (9.3 ± 0.6), with statistically significant differences (p = 0.008) (Table 2).

## Summary of Findings:

[1] Zirconia abutments resulted in the most favorable peri-implant tissue health, with significantly lower probing depths, bleeding on probing, and plaque index compared to titanium and gold alloy abutments.

[2] In terms of esthetic outcomes, zirconia abutments also outperformed titanium and gold alloy abutments, as reflected in higher PES and WES scores.

## Discussion:

The results of this study demonstrate that abutment material significantly influences both peri-implant tissue health and esthetic outcomes in fixed prosthodontics. Specifically, zirconia abutments showed superior performance in terms of clinical parameters and esthetic scores compared to titanium and gold alloy abutments. Zirconia abutments exhibited the lowest probing depths and the most favorable soft tissue response, which is consistent with other studies that report improved peri-implant tissue health with zirconia compared to metallic abutments [1]. The lower probing depth associated with zirconia may be due to its favorable tissue integration properties and reduced bacterial adhesion [2]. This finding aligns with Degidi et al. who found that zirconia surfaces tend to accumulate less plaque, which can contribute to lower inflammation and peri-implant tissue breakdown [3]. The superior bleeding on probing (BOP) results for zirconia abutments in this study are in agreement with previous research indicating that zirconia's biocompatibility and smoother surface promote less inflammatory response in the peri-implant soft tissues [4]. Other studies have also shown that zirconia's high hydrophilicity may play a role in supporting healthy soft tissue attachment and reducing peri-implant inflammation [5]. The lower BOP scores for zirconia, as seen in this study, are comparable to findings by Kajiwara et al. who observed minimal inflammatory response with zirconia in clinical settings [6].

The plaque index (PI) in the zirconia group was significantly lower than in the titanium group, which concurs with studies showing that zirconia surfaces are less prone to bacterial colonization compared to titanium [7]. Plaque formation is a key determinant of peri-implantitis, and the lower plaque accumulation on zirconia abutments could explain the better clinical outcomes observed in this study [8]. Findings by Rimondini *et al.* confirm that the smoothness of zirconia abutments reduces plaque formation, further supporting the results of this study [9]. In terms of peri-implant soft tissue thickness, zirconia abutments showed a significant increase compared to titanium and gold alloy abutments. This could be attributed to the fact that zirconia promotes better soft tissue adherence and stability [10]. The increased tissue thickness observed in this study has been similarly reported by Canullo *et al.* who noted that zirconia abutments positively influence mucosal dimensions [11]. The thicker soft tissues around zirconia abutments not only improve tissue health but also enhance the esthetic outcome by reducing the risk of abutment shine-through, particularly in patients with thin gingival biotypes [12]. Esthetic outcomes, as measured by the Pink Esthetic Score (PES) and White Esthetic Score (WES), were also superior for zirconia abutments. The higher PES observed with zirconia abutments is consistent with other research highlighting the ability of zirconia to support optimal gingival contour and coloration, which is particularly important in anterior restorations [13]. Studies by Jung *et al.* support the finding that zirconia abutments are more esthetically pleasing due to their tooth-like color and translucency, which contributes to the higher WES scores observed in this study [4]. Furthermore, zirconia's ability to prevent soft tissue discoloration compared to titanium has been well-documented in the literature, further supporting its use in esthetically demanding cases [5].

The findings regarding titanium abutments align with prior research that acknowledges titanium's strong mechanical properties and biocompatibility, but notes potential esthetic limitations, particularly in cases of thin gingival tissue [6]. Titanium's tendency to cause a grayish discoloration of the peri-implant mucosa has been a recognized challenge in achieving optimal esthetic outcomes [7]. While titanium abutments continue to be widely used due to their strength and long-term success, the esthetic limitations compared to zirconia are becoming increasingly apparent [8]. Gold alloy abutments, while not as frequently used in contemporary prosthodontics, performed adequately in this study, particularly in terms of tissue response and esthetic scores. Studies have shown that gold alloy abutments can support good soft tissue health, likely due to their smooth surface and resistance to bacterial adhesion [9]. However, gold alloy abutments are less esthetically pleasing than zirconia due to their metallic appearance, which can be a limiting factor in anterior restorations [10]. The slightly lower PES and WES scores for gold alloy abutments observed in this study are consistent with other studies that have noted their limitations in achieving ideal esthetic results compared to more modern ceramic materials [11].

The overall superior performance of zirconia abutments in this study is supported by a growing body of literature that emphasizes the benefits of zirconia in both peri-implant health and esthetic outcomes [12]. Zirconia abutments not only provide excellent soft tissue integration and minimal inflammatory response, but also meet the high esthetic demands of patients, particularly in the anterior zone [13]. Given the clinical and esthetic advantages observed in this study, zirconia abutments may be the material of choice for many fixed prosthodontic cases, especially where esthetics are a primary concern [4, 5].

## Conclusion:

Zirconia abutments demonstrated superior peri-implant tissue health and esthetic outcomes compared to titanium and gold alloy abutments in this study. These findings suggest that zirconia is a highly suitable material for both functional and esthetic success in implant-supported prostheses, particularly in cases requiring high esthetic demand. Future long-term studies are necessary to further validate the findings and explore the performance of zirconia abutments over extended follow-up periods.

## Figures and Tables

**Table 1:** Comparison of clinical parameters among different abutment materials at 12 months

Clinical Parameter	**Titanium Abutments (n=30)**	**Zirconia Abutments (n=30)**	**Gold Alloy Abutments (n=30)**	**p-value**
Probing Depth (mm)	2.2 ± 0.4	1.8 ± 0.3	2.0 ± 0.5	0.035*
Bleeding on Probing (%)	10%	5%	8%	0.048*
Plaque Index	0.9 ± 0.2	0.6 ± 0.1	0.7 ± 0.2	0.021*
Soft Tissue Thickness (mm)	2.4 ± 0.3	2.8 ± 0.4	2.5 ± 0.2	0.041*

**Table 2:** Esthetic outcomes (PES and WES) at 12 months

**Esthetic Parameter**	**Titanium Abutments (n=30)**	**Zirconia Abutments (n=30)**	**Gold Alloy Abutments (n=30)**	**p-value**
Pink Esthetic Score (PES)	9.2 ± 0.8	11.0 ± 0.6	10.5 ± 0.7	0.012*
White Esthetic Score (WES)	8.5 ± 0.5	9.8 ± 0.4	9.3 ± 0.6	0.008*
